# Cultural adaptation, content, and protocol of a feasibility study of school-based “Let’s learn about emotions” intervention for Finnish primary school children

**DOI:** 10.3389/fpsyt.2023.1334282

**Published:** 2024-01-11

**Authors:** A. Sourander, S. Ishikawa, T. Ståhlberg, K. Kishida, Y. Mori, K. Matsubara, X. Zhang, N. Hida, T. Korpilahti-Leino, T. Ristkari, S. Torii, S. Gilbert, S. Hinkka-Yli-Salomäki, H. Savolainen, V. Närhi

**Affiliations:** ^1^Research Centre for Child Psychiatry, University of Turku, Turku, Finland; ^2^INVEST Flagship Centre, University of Turku, Turku, Finland; ^3^Department for Child Psychiatry, Turku University Hospital, Turku, Finland; ^4^Faculty of Psychology, Doshisha University, Kyoto, Japan; ^5^Department for Adolescent Psychiatry, Turku University Hospital, Turku, Finland; ^6^School of Humanities, Kwansei Gakuin University, Nishinomiya, Japan; ^7^Japan Society for the Promotion of Science, Tokyo, Japan; ^8^Organization for Research Initiatives and Development, Doshisha University, Kyoto, Japan; ^9^Center for Wing of Empirically Supported Treatments, Doshisha University, Kyoto, Japan; ^10^School of Educational Sciences and Psychology, University of Eastern Finland, Kuopio, Finland; ^11^Department of Education, University of Jyväskylä, Jyväskylä, Finland

**Keywords:** school-based interventions, early interventions, socio-emotional skills, cultural adaptation, cognitive behavioral therapy, study protocol

## Abstract

**Introduction:**

Emotional awareness and emotion regulation are crucial for cognitive and socio-emotional development in children. School-based interventions on socio-emotional skills have the potential to prevent these problems and promote well-being of children. The Japanese school-based program, Universal Unified Prevention Program for Diverse Disorders (Up2-D2), has shown preventive effects on mental health of children in Japan. The aims of this protocol paper are to describe the unique process of adapting the Up2-D2 from Eastern to Western context, and to present a feasibility study of the intervention, conducted in Finland.

**Methods:**

The cultural adaptation process started with the linguistic translation of materials, followed by the modification of language to fit the Finnish context. While the Japanese ideology was saved, some content was adapted to fit Finnish school children. Further modifications were made based on feedback from pupils and teachers. The Finnish version of the program was named “Let’s learn about emotions” and consisted of 12 sessions and targeted 8- to 12-year-old pupils. A teacher education plan was established to assist Finnish teachers with the intervention, including a workshop, teachers’ manual, brief introductory videos, and online support sessions. A feasibility study involving 512 4th graders in the City of Hyvinkää, South of Finland, was conducted. It assessed emotional and behavioral problems, classroom climate, bullying, loneliness, perception of school environment, knowledge of emotional awareness, and program acceptability.

**Discussion:**

The originality of this study underlies in the East–West adaptation of a cognitive behavioral therapy-based program. If promising feasibility findings are replicated in Finland, it could pave the way for further research on implementing such programs in diverse contexts and cultures, promoting coping skills, awareness, social skills and early prevention of child mental health problems.

**Ethics:**

The ethical board of the University of Turku gave ethics approval for this research. The educational board of the City of Hyvinkää accepted this study.

## 1 Introduction

Emotional awareness and emotion regulation contribute to cognitive and socio-emotional development and are strongly related to emotional and behavioral difficulties (1). Behavioral problems serve as risk factors for later adverse outcomes, including school disengagement (2, 3). Pupils’ behavioral problems are associated with classroom climate (4), and teachers’ stress (5). School-based interventions aiming to prevent emotional and behavioral problems can be beneficial not only for children and their families but also for schools and teachers. Additionally, cost-effective prevention programs are beneficial at societal levels (6).

Schools are optimal for delivering interventions as they can reach practically all children during a critical period in their cognitive and socio-emotional development. Preventive programs executed in schools follow the ecological theory, which regards different environments as interconnective systems influencing child development (7, 8). Child’s difficulties reflect the contradiction between child’s skills and the environmental demands, and therefore preventive actions should target the entire system (9). Among the theoretical foundations of numerous school-based interventions, the ‘Social Learning Theory’ developed primarily by Bandura and Walters (10), is a key framework. This principle has served as the foundation for numerous interventions designed to shape student behaviors, attitudes, and reactions using well-planned environments and role modeling. Preventive interventions can be universal (primary), selected (secondary) and indicated (tertiary) (11). The aim should not only be the prevention of problems, but the promotion of well-being and acknowledgement of protective factors, as these contribute to building resilience (12). Teaching these skills enhances children’s potential for success socially, academically and in overall in modern life (13). The Finnish government emphasizes the emerging need for preventive measures to support the well-being of children in schools (14).

Children’s learning occurs alongside with peers and teachers. Teacher’s role is crucial when implementing school-based interventions. It has been observed that teacher-delivered preventive interventions are comparable to those which have been additionally delivered by professionals (15). However, teacher education and support need to be sufficient (16). The success of the interventions relies heavily on the implementation (17). The implementation is a multistep process, and several factors impact on these phases: individual and school-level factors such as knowledge and attitudes of the principals and teachers, student material and classroom atmosphere, as well as macro-level factors such as policies, finance, and leadership. The core is however based on the intervention itself, the delivery of the intervention, and the support for the deliverers, often referring to teachers (8).

### 1.1 Previous studies on school-based interventions for mental health and emotional well-being

Multiple school-based universal intervention programs have been developed to promote mental health and emotional well-being of young children. The majority of interventions designed to prevent common mental health difficulties are based on cognitive behavioral therapy (CBT) (18). Studies that focus on the reduction or prevention of mental health problems and disorders, have shown inconsistent results with only a few studies with strong evidence, most studies with weak evidence, and some with no effect at all (18, 19). Mental health promotion programs, such as social and emotional learning (SEL) Greenberg (20) and positive psychological interventions (PPIs) (21) are shown to be effective in improving social skills, coping skills, help-seeking skills, psychological well-being, emotional regulation, and academic performance, and reduction of emotional problems according to reviews and meta-analyses (22–25). Evidence-based interventions enable the delivery of the highest quality practices to individuals. The importance of good design and execution of interventions has been highlighted (17).

### 1.2 The original intervention Up2-D2

Many of the previous school interventions have specifically targeted anxiety, depressive symptoms, or behavioral problems (26). However, a Japanese school-based program utilized a transdiagnostic approach targeting both internalizing and externalizing problems at the same time, within the same program. This program, called the Universal Unified Prevention Program for Diverse Disorders (Up2-D2) was developed in Kyoto, Japan (27) by an interdisciplinary team consisting clinical psychologists, developmental psychologists and consulting teachers. There are several features to be noted regarding the development and feasibility studies of the program. The Up2-D2, as a universal unified school-based prevention program, consists of components mainly from CBT, but also from SEL and positive psychology. To address transdiagnostic mental health problems, the program applied a common elements approach, which involves compiling common components derived from evidence-based treatments for diverse disorders. The approach is applicable when the components are separable, independent, and structured (28). Clearly the approach is advantageous when teachers implement the program in their classroom since every class is separated by school timetable and each lesson is organized based on the predetermined curriculum. The Up2-D2 was developed based on accumulated experiences from the previous studies which were implemented by schoolteachers rather than mental health professionals (e.g., clinical psychologists, psychiatrists) for more than two decades [see Ishikawa et al. (29)].

The Up-D2 was developed based on the user-centered design (UCD) for psychosocial interventions (30). Although most previous school-based preventive interventions have focused on a single type of psychopathology, CBT approaches originally share the components between internalizing and externalizing disorders [see Society of Clinical Child and Adolescent Psychology (31)] and theoretically produce a broad paradigm treating gain [e.g. (32)]. To make the most of its gains, the Up2-D2 includes UCD concept during the development phase, in contrast to ordinary adaptation and implementation which are usually addressed after completion of efficacy studies (27). For example, it includes manga for the children to get excited and adhere to the materials to increase its learnability. The program is based on a story-like approach, by presenting and guiding three children who experience different kind of difficulties in their daily lives (i.e., anxiety, depression, and anger), and pupils can learn about different social and emotional skills through the material. Each lesson starts with an introductory manga that provides a preview of the difficulties that the characters encounter. A new skill is taught in every lesson as an “invention” (magic tools) from a “sensei” (master) of emotions, and children can learn each skill collecting all the inventions through the lessons. The program was developed to have a positive orientation, which makes the program more likely to be well-received, for both teachers and students, compared to a program targeted only at-risk students. The program also includes lower cognitive load when compared to targeted CBT-based interventions.

Feasibility studies have shown promising results in actual school settings in Japan, such as significant decreases in pupil and parent-reported mental health symptoms measured by Strengths and Difficulties Questionnaire (SDQ) (33–35). There was improvement in pupil-reported self-efficacy and teacher-reported pupils’ social skills in pre-post assessment before and immediately or 3 months after the program (35). In addition, the Up2-D2 decreased anxiety symptoms in students in 7th grade immediately after the nation-wide school closure due to COVID-19 pandemic (36). Moreover, high satisfaction, understanding, and usability of the Up2-D2 from teachers in elementary schools was obtained compared with that in secondary schools (37).

### 1.3 Cultural adaptation

The aim of cultural adaptation is to increase the adaptability and feasibility of an intervention (38). This paper indicates what adaptations are essential in the context of universal prevention programs implemented by school teachers in elementary school. Cultural adaptation can be divided into different stages: before the intervention trial, the program should be carefully planned, tested, and enhanced by the piloting results (39). Adapting a CBT-based intervention from East Asian cultural context to Western context is not common and that is the originality in this study. This is because most evidence-based psychosocial interventions for children and adolescents including CBT-based interventions originated in a Western context. Interestingly, the Up2-D2 was developed in East Asian cultural context after importing evidence-based practice from Western countries. This effort could shed light on commonalities and differences in school-based CBT-interventions between East and West. Previously fluent East–West transmission has been experienced with mindfulness-based practices (40).

The aim of this article is to (1) describe the cultural adaptation process and the content of the program as the Japanese school-based program Up2-D2 was adapted to Finnish context; (2) describe the teacher training model based on Japanese experiences but further developed to fit Finnish context; (3) provide information about a feasibility study among Finnish 4th graders (10- to 11-years-old).

## 2 Methods

### 2.1 Description of the cultural adaptation process

**Figure 1:**
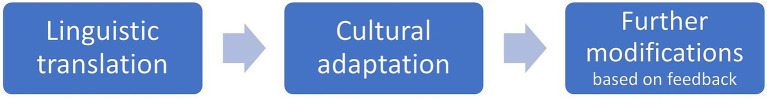
The flow diagram of the adaptation process.

Bernal et al.’s (41) ecological validity model was used as a guideline in the cultural adaptation process of the intervention. The process of adaptation was as follows (Figure 1): first, all materials from the original Japanese program (27) were translated into Finnish by two translators proficient in both Japanese and Finnish. The Finnish research team consisted of one linguist, mental health professionals (cognitive behavioral therapists, child and adolescent psychiatrists) and educational professionals. After translation, the Finnish research team modified the language to suit the target age group and CBT, and carried out cultural adaptation to ensure the content’s appropriateness for Finnish pupils and teachers. Some cultural differences were detected, such as the emphasis on politeness in the Japanese version, contrasting with the desired assertiveness in the Finnish context. Consultations with teachers and educators were conducted throughout the process. Finally, a one-lesson pilot study was conducted in two classes of 4^th^ graders (aged 10–11 years) in Turku, Finland, in May 2022. Modifications were made according to the feedback we received from pupils and teachers.

### 2.2 Content of the intervention

“Let’s learn about emotions” (Finnish name “Opitaan tunteista”) is a school-based, teacher-delivered universal program, based mainly on CBT, but also on SEL and positive psychology theories. In each lesson, evidence-based skills representing interventions (Table 1) are taught to pupils. These skills aim to enhance the emotional and behavioral regulation of the pupils and impact positively on their social skills and peer relations. The theoretical background of each lesson has been described in Table 1. Strategies include psychoeducation, behavioral activation, social skills, stress management, cognitive restructuring, gradual exposure, and problem-solving.

**Table 1:** Aims and theoretical background for each lesson.

Number of the lesson	Aim of the lesson	Theoretical framework from cognitive behavioral therapy	Sensei’s inventions (magic tools for learning socio-emotional skills)
1	Introduction and learning about emotions	Psychoeducation	Emotion sensor 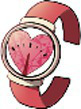
2	Exploring pleasant activities	Behavioral activation	Happy shoes 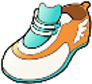
3	Friendly words	Social skills training	Friendliness potion 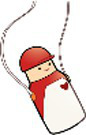
4	Assertive skills	Social skills training	Assertiveness speaker 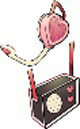
5	Relaxation techniques	Relaxation	Relaxation apple 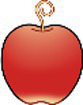
6	Identifying strengths	Strength work (from positive psychology)	Strength wound 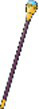
7	Discovering own cognitions	Cognitive restructuring	Thought torch 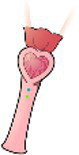
8	Challenging unhelpful thoughts	Cognitive restructuring	Thought refreshing spray 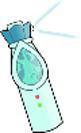
9	Preparing for gradual exposure, identifying challenges	Gradual exposure	Bravery cloak 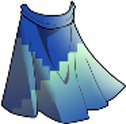
10	Creating steps for gradual exposure	Gradual exposure	Master sword 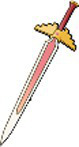
11	Problem solving skills	Problem solving	Problem solving stick 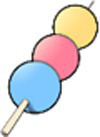
12	Review and conclusion	Repetition and practice	

Reprinted with permission from Ishikawa, Kamio (42).

**Figure 2:**
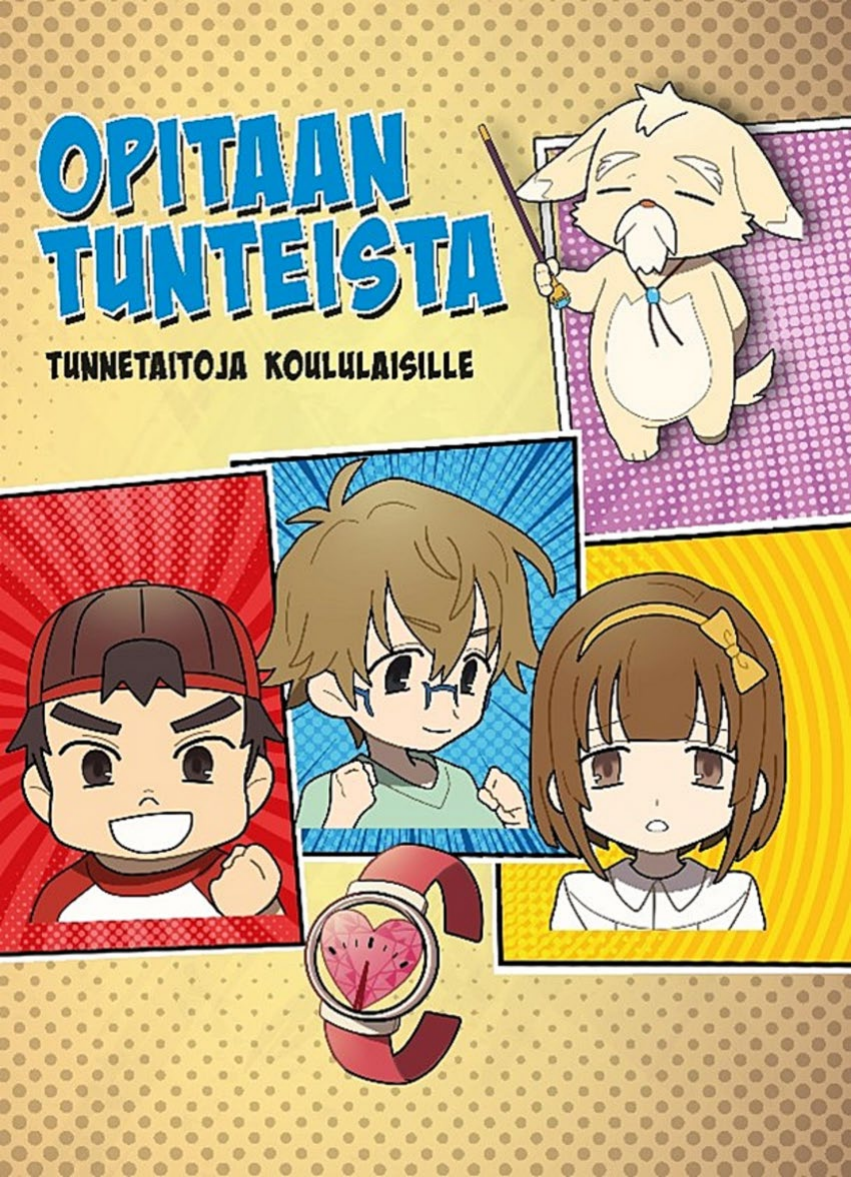
“Opitaan tunteista – tunnetaitoja koululaisille” (English: Let’s learn about emotions – emotional skills for school children): the Finnish book cover shows the three child manga characters and the master of emotions, as well as the Sensei’s first invention: emotion sensor.

The intervention is aimed to 8- to 12-year-old pupils. The program consists of 12 sessions of 45 min, preferably delivered once a week. The program features three children: Fire heart (Tulisydän), Water heart (Vesisydän), and Earth heart (Maasydän) who are 5th graders with their own characteristics and weaknesses, and a sensei/master character: Great master (Ikivanha) (Figure 2). In each lesson, the children will encounter common problems or difficulties in their lives. Guided by these examples, the topics are introduced to the pupils. The sensei will offer them “magic tools” (inventions) (Table 1), which represent new socio-emotional skills for managing difficult situations. Pupils will participate in individual and group-level writing tasks in every lesson. In addition, they will receive homework designed to help them integrate the learned content into their daily lives (e.g., Lesson 1: “Write down some situation during your week and the emotion you experienced in that situation. Rate how strong that emotion was”). A book was produced for the pupils, providing an interesting and intriguing way to study emotional skills, Figure 2.

### 2.3 Teacher training

Teachers’ education was planned to serve the needs of Finnish teachers, including a 2.5-h workshop before the intervention, a teachers’ manual, introductory videos for each lesson and four online support sessions during the spring 2023. The educational workshop contained general information of the intervention and the study, an example lesson (Lesson 1), and introduction of teachers’ manual and videos. Teachers’ manual was created based on the teachers’ materials used in Japan. The manual includes a brief theoretical description of each lesson and the aim of the lesson. Following that, the manual aligns with the pupils’ material providing practical, step-by-step instructions for each lesson. The introductory videos summarize the idea and the content of each lesson in a short and compact way. The length of each video is 1.5–2 min. Online meetings were planned to support teachers’ needs, answer questions, provide peer support comments, and summarize the following lessons. To provide comprehensive assistance to teachers, they have the option to contact the researchers via email, and if necessary, they can also access child psychiatry consultations during the entire intervention.

The aim was to design a comprehensive education and support system for teachers, providing them with sufficient materials while avoiding overburdening them. Teachers’ needs may differ between cultures and countries. Respecting teachers’ competence and autonomy are considered important in Finland. A balance between program fidelity and Finnish teachers’ autonomy was considered when planning their education.

### 2.4 Methods of the feasibility study

A single-group pretest-posttest design is used in the large ongoing feasibility study with three respondents: pupils, parents, and teachers. There are three repeated measurements, assessments are T0 at baseline, prior to the intervention; T1 immediately after the intervention; and T2 3 to 4 months after the intervention.

#### 2.4.1 Study sample and recruitment

The study was done in the City of Hyvinkää, South of Finland, to pilot the intervention for all the pupils in 4^th^ grades (aged 10–11 years) in the public elementary schools of Hyvinkää, Finland. In total 36 classes in 14 schools, total 512 4^th^ graders received the intervention implemented in their normal curriculum in spring semester 2023. All these pupils, their parents and teachers were approached with written information letter regarding the program and the study. Consent forms were administered to parents and teachers. Active assent was observed from the pupils by clearly informing them of the study and voluntary nature of the questionnaires. Each pupil was given the same education regardless of their participation in the study, but only those pupils with a parental consent were able to fill the questionnaires.

#### 2.4.2 Inclusion and exclusion criteria

Inclusion criteria were as follows: (1) pupils who currently enrolled in primary education in a public school located in Hyvinkää, Finland; (2) pupils whose parents granted active consent; (3) pupils who provided assent to participate in the study. Exclusion criteria were as follows: (1) pupils with severe intellectual disabilities; (2) pupils, whose teachers are unable to teach in Finnish and (3) pupils with a very limited understanding of Finnish.

#### 2.4.3 Data collection

Questionnaires for pupils and teachers were self-administered paper questionnaires that were filled in at schools during lessons. Teachers sent an electronic message to each parent, containing a link to university’s secure website. This website contains the information letter, consent form, and the questionnaires.

#### 2.4.4 Outcome assessments

Table 2 presents a detailed overview of the measures used at each assessment point, T0, T1 and T2. Sociodemographic information was collected from pupils (first name, birth month, school, class, gender, and first language), teachers (name, age, gender, class and school), and parents (child’s first name, birth month, class and school).

**Table 2:** Overview of measures.

Variable name	Instrument	Number of questions	Time of assessment		
			T0	T1	T2
Children					
Emotional and behavioral problems	SDQ	25 + 5	✔	✔	✔
Classroom behavioral climate	CBC	8	✔	✔	✔
Loneliness	CDI	2	✔	✔	✔
Bullying experience		8	✔	✔	✔
Perceptions of school environment		7	✔	✔	✔
Knowledge		11		✔	
Acceptability		10		✔	
Teachers					
Classroom behavioral climate	CBC	8	✔	✔	✔
Fidelity		6		✔	
Acceptability		29		✔	
Parents					
Emotional and behavioral problems	SDQ	25 + 5	✔	✔	✔
Loneliness and friendship	CDI	2	✔	✔	✔
Acceptability		8		✔	

T0, At baseline; T1, after the intervention; T2, 3–4 months after the intervention; SDQ, Strength and Difficulties Questionnaire; CBC, Classroom Behavioral Climate; CDI, Children’s Depression Inventory.

#### 2.4.5 Primary outcome

The primary outcome of this study is pupils’ emotional and behavioral problems following the original study of the Japanese version of this intervention conducted by Oka et al. (35). These emotional and behavioral difficulties were assessed by the self-report, and parent-report version of the Strengths and Difficulties Questionnaire (SDQ) (33). The SDQ is one of the most widely used screening instruments for identifying these difficulties in children and adolescents, with good internal consistency, reliability, and inter-rater agreement in school-aged children (43, 44). The Finnish version of SDQ has also been found to be reasonably reliable among school-aged children, Cronbach’s alfa being 0.71 for total score (45). The SDQ consists of five subscales measuring emotional symptoms, conduct problems, hyperactivity, peer problems and prosocial behavior. The subscales have Cronbach’s alfas varying from 0.57 for conduct disorders to 0.69 for emotional symptoms and prosocial scale among Finnish children (45). Each subscale has five items, each of which is rated 0, 1 or 2 points in accordance with being “absolutely true,” “somewhat true” or “not true.” The total difficulties score ranges from 0 to 40 (excluding prosocial scale which is scored inversely).

#### 2.4.6 Secondary outcomes

To assess the *classroom behavioral climate* (CBC), we use a shortened version of a Finnish instrument, CBC, which has adequate reliability (46, 47). Pupil and teacher reports of CBC were used in this study. *Traditional and cyber bullying and victimization* were assessed by pupils (48). Seven questions assessed *children’s perception about the school environment* (48). *Children’s loneliness* questions for pupils and parents and *Friendships* questions for parents have been modified from the Children’s Depression Inventory (CDI) scale (49, 50). Children’s self-reported *knowledge* of emotional awareness was assessed after 12^th^ lesson by using a questionnaire created for the purposes of this research by the Finnish research team. The questions test children’s learning and comprehension of the main points of the intervention. Symptoms of depression, anxiety, or conduct problems were not measured separately. Since the intervention is universal, we aimed to measure general outcomes across the whole sample rather than focusing on psychiatric symptoms, which would only have been present in some pupils. SDQ was considered to capture both internalizing and externalizing problems sufficiently.

*Acceptability and satisfaction* of the intervention were measured post-test (T1) from pupils and teachers using a short questionnaire specially developed for this study, based on the questionnaire used in the Japanese study (35) and Client Satisfaction Questionnaire by Attkisson (51).

*Fidelity* was assessed with questions for the teachers after each lesson by anonymous fidelity and satisfaction questionnaires, with six questions at T1 as well as through the observation of random lessons on-site. Members of the study group will observe the teaching passively, filling a fidelity checklist. Feasibility is measured based on the proportion of eligible participants who are approached and provide consent to enter the study, the number of dropouts at each assessment point, and the presence of missing items on outcome measures.

#### 2.4.7 Data management

Microsoft Access input forms were used in the data entry process of the children’s and teachers paper questionnaires. Parental reports were filled online on a secure site. All data is stored in protected fileservers of Turku University. These files are secured by daily backups and limited access. The access will be granted for researchers, statisticians, and data managers. After the data collection and entry phase, the data will be anonymized, validated, linked and imported to SAS datasets for statistical analyses.

#### 2.4.8 Statistical analyses

Demographic data will be presented for pupils, parents and teachers separately. Categorical variables will be presented as counts and percentages and continuous variables as means and standard deviations (SD). Outcome variables will be analyzed with linear mixed-effects models with repeated measurements. The respondents (pupil, teacher, parent) will be included in the model as a random effect, if feasible. Changes will be examined for each respondent, for each class, as well as for the total population. Linear contrasts will be used to estimate the changes of each outcome variable from baseline. The five subscales of SDQ will be analyzed separately, and each item the secondary outcome measures will be observed separately. A two-sided value of p of <0.05 will be considered significant.

### 2.5 Schedule of the study

The intervention was carried out in spring semester 2023. Collection of T0 data was conducted in February 2023, T1 in May and T2 in September 2023.

## 3 Discussion

This paper details the cultural adaptation and content formulation of a universal school-based intervention centered on socio-emotional skills. The challenge was in adapting the intervention from an Eastern context to suit a Western one. Notably, while the content was tailored to serve Finnish pupils, the distinctive Japanese visual features, which garnered admiration, were preserved.

Within the broader discourse on cognitive-behavioral therapeutic interventions, an atypical trajectory surfaces in this study: the adaptation of a framework that was developed in East Asian culture to fit a Western school environment. Typically, evidence-based psychosocial strategies, particularly those devised for children and adolescents, develop from Western paradigms. This paper describes a reversal from traditional cultural adaptation approaches and provides a way for evaluating school-based CBT methods across both Eastern and Western contexts.

Child mental health problems are one of the key challenges in public health. Providing children with evidence-based early interventions to tackle these challenges has uttermost significance for society. The possibility of the school environment in providing skills training for children on how to cope with and manage emotional and behavioral problems has not been thoroughly studied. Most school programs focused on some specific problem such as bullying. The “Lets Learn about Emotions” program is universal and transdiagnostic, covering a wide range of mental health problems. The program is based on solid theoretical bases, including CBT and positive psychology theories. Evidence-based skills are taught during 12-week curriculum-based intervention. These skills enhance emotional and behavioral regulation in pupils and impact positively on their social skills and peer relations. Teachers play a key role in skills training, providing strong face-to-safe and remote support using workshops, consultations and different types of materials.

There are several limitations to be discussed in the study. Although the program consists of SEL components, it is not a comprehensive SEL program. Participation of parents and community in this intervention was limited. This could be addressed in the future by providing more information and involvement for parents, entire schools and community. Another limitation concerns fidelity. Whereas we examined fidelity by teacher reports and observation, we did not include pupil’s perspective. Fidelity comprises also the attitudes and how teachers model the skills from the program in their own behavior. These limitations can be exploited in the future research. Fidelity checks could also be built in the program itself in the future.

Teaching emotional and social skills is educating the children for life, rather than aiming only for academic achievements. Schools offer an ideal place to provide a universal program aiming at improving these skills. The present study provides information about the feasibility of teaching emotional and social skills in elementary school settings. This implicitly challenges the current role of school system, which primarily aims to teach academic skills, by additionally educating children to cope with emotional and behavioral problems. If the promising feasibility findings reported in the original Japanese study are replicated in Finland, this will give strong reasoning for further efficacy and implementation research both in Western and non-Western societies, aiming to enhance coping skills, awareness, and early prevention of child mental health problems in a real-world context.

## Ethics statement

The studies involving humans were approved by the Ethics Committee for Human Sciences at the University of Turku. The studies were conducted in accordance with the local legislation and institutional requirements. Written informed consent for participation in this study was provided by the participants’ legal guardians/next of kin.

## Author contributions

AS: Conceptualization, Funding acquisition, Methodology, Project administration, Resources, Supervision, Writing – original draft, Writing – review & editing, Validation. SI: Conceptualization, Methodology, Project administration, Resources, Supervision, Writing – original draft, Writing – review & editing, Validation. TS: Investigation, Methodology, Project administration, Writing – original draft, Writing – review & editing, Validation. KK: Resources, Writing – review & editing, Validation. YM: Methodology, Project administration, Validation, Writing – original draft, Writing – review & editing. KM: Resources, Validation, Writing – review & editing. XZ: Writing – review & editing NH: Resources, Validation, Writing – review & editing. TK-L: Methodology, Validation, Writing – review & editing. TR: Methodology, Validation, Writing – review & editing. ST: Validation, Writing – review & editing. SG: Methodology, Writing – review & editing. SH-Y-S: Data curation, Methodology, Resources, Writing – review & editing. HS: Methodology, Supervision, Writing – review & editing. VN: Methodology, Supervision, Writing – review & editing.
